# Introgression of Physiological Traits for a Comprehensive Improvement of Drought Adaptation in Crop Plants

**DOI:** 10.3389/fchem.2018.00092

**Published:** 2018-04-10

**Authors:** Sheshshayee M. Sreeman, Preethi Vijayaraghavareddy, Rohini Sreevathsa, Sowmya Rajendrareddy, Smitharani Arakesh, Pooja Bharti, Prathibha Dharmappa, Raju Soolanayakanahally

**Affiliations:** ^1^Department of Crop Physiology, University of Agricultural Sciences, Bengaluru, India; ^2^ICAR-National Research Centre for Plant Biotechnology, New Delhi, India; ^3^Saskatoon Research and Development Centre, Agriculture and Agri-Food Canada, Saskatoon, SK, Canada

**Keywords:** drought adaptive traits, water productivity, water-use efficiency, physiological breeding, carbon isotope discrimination, cellular level tolerance, transgenics

## Abstract

Burgeoning population growth, industrial demand, and the predicted global climate change resulting in erratic monsoon rains are expected to severely limit fresh water availability for agriculture both in irrigated and rainfed ecosystems. In order to remain food and nutrient secure, agriculture research needs to focus on devising strategies to save water in irrigated conditions and to develop superior cultivars with improved water productivity to sustain yield under rainfed conditions. Recent opinions accruing in the scientific literature strongly favor the adoption of a “*trait based*” crop improvement approach for increasing water productivity. Traits associated with maintenance of positive tissue turgor and maintenance of increased carbon assimilation are regarded as most relevant to improve crop growth rates under water limiting conditions and to enhance water productivity. The advent of several water saving agronomic practices notwithstanding, a genetic enhancement strategy of introgressing distinct physiological, morphological, and cellular mechanisms on to a single elite genetic background is essential for achieving a comprehensive improvement in drought adaptation in crop plants. The significant progress made in genomics, though would provide the necessary impetus, a clear understanding of the “traits” to be introgressed is the most essential need of the hour. Water uptake by a better root architecture, water conservation by preventing unproductive transpiration are crucial for maintaining positive tissue water relations. Improved carbon assimilation associated with carboxylation capacity and mesophyll conductance is important in sustaining crop growth rates under water limited conditions. Besides these major traits, we summarize the available information in literature on classifying various drought adaptive traits. We provide evidences that Water-Use Efficiency when introgressed with moderately higher transpiration, would significantly enhance growth rates and water productivity in rice through an improved photosynthetic capacity.

## Introduction

Water can be considered as one of the major factors that helped the nomadic hunter-gatherers to settle down in civilized societies. All ancient civilizations flourished in river basins with an assured source of water throughout the year (Gupta, 2004). The progress in the domestication of plant species for human consumption, peace, and prosperity of human settlements, can be attributed to water. Although irrigation requirements alone account for more than 60% of all fresh water used, the ever increasing human populations is quickly leading to an escalation in civic and industrial demand for fresh water. This scenario of withdrawal of water away from agriculture triggered by erratic monsoons due to the ensuing global climate change and hence food production is expected to be severely affected (Yusuf and Christina, 2008; Finlayson, 2014).

Nevertheless, providing food and nutrient security to an increasing human population remains the major, if not the sole, responsibility of the agriculture research. Unlike during the green revolution era, agriculture research now faces an unprecedented challenge of producing “*more*” food with “*less*” resources, especially water. In this context, the slogan “*more crop per drop*” has the greatest relevance. Therefore, agriculture research should focus on devising strategies for saving water under the irrigated system and to improve productivity under rainfed dry land conditions.

Agriculture in the tropical and sub-tropical climates is characterized by regions with extreme conditions of water availability. In “command” areas adjacent to dams there is an abundance of water, while in the distant “tail-end” areas severe water scarcity is noticed seasonally. Thus, there is a substantial “Rainfed” area where water scarcity is severe. Several water management strategies and agronomic practices have been developed to conserve soil moisture. Mulching the soil with plant debris and biodegradable plastic (Prosdocimia et al., 2016) have long been developed and are being practiced. These water conservation approaches have significantly contributed to improved productivity in dry land crops like sorghum (Unger and Jones, 1980), groundnut (Ramakrishna et al., 2005), and soyabean (Arora et al., 2011).

Water management agronomy, though highly relevant, demands adoption of informed agriculture. Furthermore, it requires the commitment from the farmer to implement the precisely monitored schedules which, at times, can be nearly impossible. Besides its inherent difficulties of water conservation agronomy, it appears that the water management options have attained their potentials. Possibly, we need to reduce the water required for a given productivity under irrigated conditions and to sustain productivity under rainfed ecosystem by enhancing carbon assimilation capacity even with reduced stomatal conductance. Hence, the potential physiological processes hold in further improving water productivity and drought adaptation is infinite (Parry et al., 2005; Flexas, 2016). From the agronomic perspective, drought adaptation is linked with sustained productivity even under water limited conditions. Phenomenal progress over the last couple of decades has provided excellent leads in terms of understanding the response of plants to drought stress and various mechanisms associated with drought adaptation. For a comprehensive improvement in drought adaptation, several relevant traits must be introgressed on to a single elite genetic background (Araus et al., 2008; Reynolds and Tuberosa, 2008; Raju et al., 2014). This emphasized on the need to identify specific traits that would improve drought adaptation, establish their relevance and develop approaches to phenotype them to capture genetic variability.

Hence forward, crop improvement needs to conceptually focus on genetic enhancement with reduced water requirement for a given yield potential. In this review, we describe the relevance of genetic enhancement of crop cultivars to improve productivity under water limiting conditions. We discuss the possible physiological and molecular mechanisms that need to be considered for achieving improved drought adaptation. Among several traits, those associated with maintenance of leaf turgor and carbon metabolism have the greatest relevance in sustaining growth rates under drought. Despite the knowledge on traits, the progress in breeding for improved water productivity has been limited. We make an attempt to evaluate the reasons for the lack of success in improving crop productivity and suggest possible approaches to overcome this lacuna in genetic enhancement.

## Genetic enhancement: drought tolerant variety vs. drought adaptive trait

Progress made in understanding drought adaptive mechanisms led to their classification as drought escape, dehydration postponement, and desiccation tolerance (Turner, 1986; Khan et al., 2010). With rapid phenological development and completion of life cycle before the onset of drought, plant can escape stress effects. On the other hand, maintenance of tissue turgor through improved water uptake and water conservation strategies would delay the onset of stress to plants. Maintenance of positive carbon gain and other metabolism even under decreasing leaf tissue turgor is referred to as “desiccation tolerance.” Each of these drought adaptive strategies have specific relevance to survival of plants under drought condition. But, from the agronomic point of view, any genotype that accumulates relatively more biomass even under reducing soil moisture conditions is considered as drought adaptive (Levitt, 1973). This definition clearly emphasizes the relevance of maintaining crop growth rates under receding soil moisture conditions and has been the most widely adopted strategy to select superior genotypes (Richards, 1996). Breeders thus adopted selecting for higher absolute yields under stress as a crop improvement strategy which undoubtedly led to a major breakthrough in yield in the late 70's (Venuprasad et al., 2007). However, at present, the perceivable yield gain through this selection strategy is less than 1% annually (Fischer et al., 2014).

Crop improvement in the past relied on creating random recombination followed by a selection for higher yields. Breeders tended to call these varieties “drought tolerant” as they produce relatively higher yields under stress. This strategy in the current scenario, is predicted to significantly fall short of the targeted yields needed to feed world populations by 2050 (Ray et al., 2013). A narrow variability in absolute yields among the already improved high yielding cultivars, a high genotype and environment (G × E) interaction for yield and low heritability have been attributed to be major factors that may limit further crop improvement through selection for yield *per-se* (Araus et al., 2008; Reynolds and Tuberosa, 2008). On the other hand, component physiological traits are more stable across environments and hence have greater breeding value (Reynolds and Langridge, 2016). A greater sustainability of rice yield was noticed when diverse traits were used in the selection strategy (Raju et al., 2014). Therefore, understanding the mechanisms of drought adaptation at a component trait level becomes essential to break this yield barrier as well as to improve productivity in resource limiting environments. These recent developments strongly emphasize on the relevance of adopting a trait based breeding approach to enhance productivity under drought stress conditions over the strategy of selecting for yield *per-se* under drought (Reynolds et al., 2012). Therefore, breeding to improve specific drought adaptive traits is expected to be mere rewarding than a mere selection for drought adaptive genotypes. However, progress has been generally slow.

## Why genetic enhancement for drought adaptation is slow and how to accelerate?

The impediment for improving productivity under water limiting conditions comes mainly from two factors, viz., the complexity of drought and the equally complex responses of plants to stress. The complexity of drought in turn arises because of a large array of influences it has on plant growth, metabolism, and development which can remarkably vary depending on the time of occurrence and the severity of drought stress. All plant development processes starting from germination, seedling establishment, vegetative, flowering to grain filling phases, are quite sensitive to drought stress (Alberte et al., 1975; Centritto et al., 2009; Farooq et al., 2014), which only illustrates the complexity of drought and drought stress responses of plants.

Though drought stress can strain plant growth at any phenological stage/s, all these complex effects culminate in reducing crop yield (Daryanto et al., 2017). Furthermore, growth stages show varied sensitivity to stress in diverse crop species. Therefore, improving stress tolerance in any one of the stages of plant development would severely fall short of comprehensively improving yield under stress. Because of the multitude of responses of plants to stress, a single trait may not provide higher levels of tolerance. In a recent review, Flexas (2016) opined that the slow progress in genetic enhancement under drought has been primarily because single trait or gene is being used at a time by researchers. Though this strategy is scientifically valid, for a more comprehensive genetic enhancement to improve drought adaptation of field crops, an approach that involves a simultaneous improvement in multiple traits/mechanisms needs to be adopted (Sheshshayee et al., 2013). Therefore, it is apparent that several independent traits need to be introgressed on to a single elite genetic background to achieve improved performance under stress. Despite the tremendous progress in genomic approaches leading to the discovery of genes and QTLs, and evolution of robust methodology for transferring these genes and QTL to a recipient background, the progress in genetic enhancement has still remained nominal. Therefore, to overcome this impasse in crop improvement, it is essential to develop deeper understanding on the specific traits that confer tolerance to stress at a specific growth stage or to a specific metabolic or cellular function.

Various adaptive traits that the physiologists have long been suggesting are classified into several categories. Based on the level of organization and their relevance, drought adaptive traits are often classified into “*constitutive*” and “*acquired*” tolerance traits (Table 1). Constitutive traits are those that are present all through the life of a plant and have relevance at all times. Traits such as roots, stomata, leaf thickness etc. have specific influence on the growth of plants on a time integrated scale. These constitutive traits display a higher level of stress adaptation and functional conservation across environments. For instance, the stress adaptation provided by deep rootedness would have greater adaptability in diverse environments (Uga et al., 2013; Tardieu et al., 2017). Some of these traits will have specific influence on crop performance on a long time scale. The traits such as Harvest Index, yield, total biomass etc. are as a result of several traits/mechanism all through the life of plants. These traits ultimately have an influence on Drought susceptibility index (DSI) and hence are referred to an “integral traits.”

**Table 1:** Classification of traits based on the level and time of their expression and their dissection to sub-components.

**Traits**	**References**
**CONSTITUTIVE TRAITS**	
Phenology	Blum, 1996
Waxes	Manavalan and Nguyen, 2012
Stomatal density	Kholová et al., 2010
Water-Use Efficiency (WUE)	Blum, 1996
Specific Leaf Nitrogen (SLN)	Agrama et al., 1999
Specific Leaf Weight (SLW- anatomical trait)	Blum, 1996
Leaf pigments/stay green	Lopes and Reynolds, 2012
Roots	Blum, 1996
Plant height	Kamoshita et al., 2008
Leaf area	Raju et al., 2016
**ACQUIRED TOLERANCE TRAITS**	
Osmolytes synthesis	Kamoshita et al., 2008
Osmotic adjustment	Basu et al., 2016
Cell viability	Kamoshita et al., 2008
Cell membrane stability	Kamoshita et al., 2008
Chlorophyll stability	Kamoshita et al., 2008
Protein synthesis	Kamoshita et al., 2008
Stress induced changes in gas exchange	Kamoshita et al., 2008
Scavenging systems	Kamoshita et al., 2008
Cytotoxic compounds	Kamoshita et al., 2008
Changes in growth hormones (ABA)	Monneveux et al., 2012
Water status/relations	Kamoshita et al., 2008
Canopy temperature	Kamoshita et al., 2008
Leaf senescence	Kamoshita et al., 2008
Leaf rolling/folding	Monneveux et al., 2012
Flowering time alterations	Kamoshita et al., 2008
**INTEGRAL TRAITS**	
Drought Susceptibility Index (DSI)	Kamoshita et al., 2008
Survival & recovery	Kamoshita et al., 2008
Yield & Harvest Index	Kamoshita et al., 2008
Remobilization of resources	Monneveux et al., 2012
Net Assimilation Rate (NAR)	Araus et al., 2002

On the other hand, “acquired traits” are strongly expressed only when plant experience stress (Sung et al., 2003). These acquired tolerance traits are governed by the coordinated gene expression that delivers gene products when needed at the appropriate growth stage/metabolic status or in response to specific environmental cues (Schmid et al., 2005; Wang et al., 2010). Though a large number of mechanisms are known to contribute to acquired tolerance, the emphasis, however, has been on oxidative stress tolerance mechanisms, osmotic adjustment, and a few aspects associated with protein turnover (Vinocur and Altman, 2005). Some of the important sub-component traits of acquired tolerance are given in Table 1. Expression of specific genes governing these mechanisms brings in altered metabolism for adaptation. It is also demonstrated that the level of expression of these acquired traits vary considerably among genotypes hence are responsible for the observed genetic variability. These “acquired tolerance” traits illustrate the propensity of a genotype to respond to stress and hence its ability to survive stress period and recover on stress alleviation. In this context, these acquired traits would also have a strong influence on survival under drought and recovery of growth on stress alleviation.

In summary, crop growth and productivity under stress is governed by both constitutive/integral traits as well as the acquired tolerance traits. It has been shows that the advantage of constitutive traits in improving drought adaptation is significantly enhanced in the genetic background of a genotype with reasonably higher levels of acquired tolerance. Some of the important constitutive and acquired traits are indicated in Table 1.

**Figure 1:**
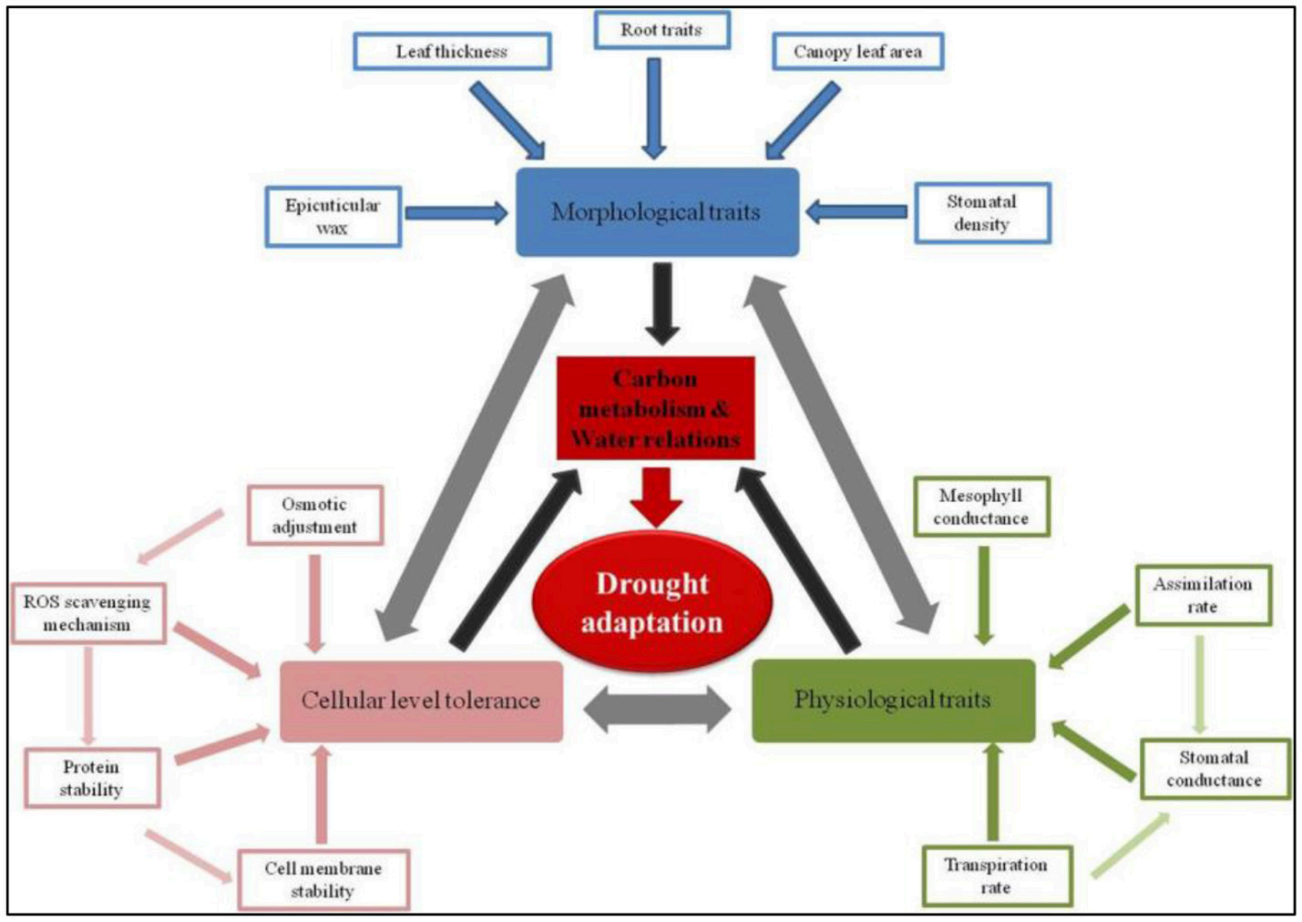
Dynamic classification of drought adaptation—Specific traits governing physiological, morphological, and cellular processes. Drought adaptation is a cascade of several physiological, morphological, and cellular mechanisms. A significant degree of interdependence between these mechanisms exists as illustrated by the double headed arrows. These physiological, morphological, and cellular mechanisms in turn are governed by a number of component traits which could be constitutive and/or acquired traits (refer Table 1). These mechanisms collectively lead to the maintenance of positive carbon gain and tissue turgor (black arrows). Thus, from an agronomic point of view, a comprehensive drought adaptation is possible only when any trait contributes to the maintenance of carbon assimilation and water relations (red arrow).

More dynamically, comprehensive drought adaptation can be visualized as a phenomenon arising through the maintenance of tissue water relations and positive carbon gain. Several morphological, physiological, and/or cellular mechanisms converge to help maintain positive carbon gain and tissue turgor. A myriad of specific traits in turn influence the relevance of these morphological, physiological, and cellular mechanisms that ultimately improve the plants ability to adapt to drought conditions. A few very important component traits contributing to drought adaptation are illustrated in Figure 1. For instance, morphological traits such as roots and leaf surface characters like wax deposition, pubescence etc., respectively influence water uptake from deeper soil profiles and water conservation by reducing heat load on the leaf. These mechanisms that help maintain tissue turgor in turn contribute to the maintenance of relatively higher positive carbon gain. Similarly, cellular level mechanisms that help maintaining cell membrane integrity through better management of reactive oxygen species (ROS) would support cell expansion and metabolism. Furthermore, these cellular mechanisms can also contribute to improved metabolism even under conditions of reducing tissue turgor. Though the scenario of improving drought adaptation may appear complex, in reality it can be simplified to a few very important constitutive and acquired tolerance traits (also listed in Table 1). Improving any these specific trait would lead to improved drought adaptation. But for a more comprehensive improvement, it appears that several of these traits should operate in unison (Daryanto et al., 2017). In the following sections, we discuss various possibility of combining these diverse traits to demonstrate improved drought adaptation.

A deeper understanding of physiological mechanisms associated with drought adaptation led to trait dissection and deployment in crop improvement (Reynolds and Tuberosa, 2008; Chandra, 2010; Tuberosa, 2012; Araus and Cairns, 2014). This effort was strongly complemented by an extensive improvement in high throughput phenotyping efforts (Araus and Cairns, 2014; Kuijken et al., 2015; Vadez et al., 2015) which have led to the initiation of focused breeding for physiological traits (Tardieu et al., 2017). Development of imaging based technology has further strengthened the phenotyping efforts by bringing in much higher precision with enhanced throughput to screen large populations (Yang et al., 2013). Therefore, the future crop improvement efforts are expected to rely strongly on approaches that combine the expertise in plant physiology, molecular biology, and genomics including computational biology to evolve a molecular breeding strategy (Mir et al., 2012; Gaspar et al., 2013; Reynolds and Langridge, 2016). These developments have significantly enhanced the optimism in accelerating breeding process to improve productivity under drought. The major emphasis has therefore shifted to understanding the mechanisms of resource capture and the efficient use of the captured resources.

## Are resource capture and use efficiency relevant traits?

With the consensus that a trait based genetic enhancement can generate sustained productivity under water limited conditions, the focus is therefore being concentrated on identifying the most appropriate combination of traits. Since the major goal is to improve productivity under stress, survival strategies such as drought escape and dehydration postponement are not very useful as most of these traits are often counter-productive (Turner et al., 2006). Therefore, identifying traits that enhance growth and productivity even under drought have greatest relevance. It is well-known that the boost in productivity achieved during Green Revolution was predominantly possible through maximizing resource capture (Andersen et al., 1985). Genotypes that were highly responsive to added fertilizers and irrigation were favored in breeder's selection. The semi-dwarf phenotype in cereals with erect canopy architecture ensured a better distribution of light and hence maximized light energy capture (Matthew et al., 2000). Though dramatic success in crop yields was witnessed due to these factors, it is widely believed that the selection did not favor resource-use efficiencies. Therefore, crop improvement for resource limiting conditions must focus on improving efficiency of using resources like light, nutrients (Sinclair and Horie, 1987), and most importantly water (Passioura, 1983; Blum, 2009).

Radiation and nutrient use efficiencies are generally accepted as useful traits that enhance growth rates and yield potentials. However, the relevance of WUE has been a topic of fierce debate (Blum, 2009, 2011). The growth model of Passioura (1983) emphasized that biomass accumulation is a function of water use and WUE. This “Pedagogical” model has long been a topic of intense discussion in the literature. It is unequivocally demonstrated that total water use is strongly related to crop growth rates and total biomass accumulation (Impa et al., 2005; Xin et al., 2009). Accordingly, any trait that governs total water use would also be associated with crop growth rates. However, higher transpirational water use is not a feasible strategy under water limited conditions. Therefore, decreasing transpiration emerged as an automatic choice to improve water economy. Evolutionally also, reduction in transpiration was found to be a survival strategy under water limited conditions. This conservative strategy from the agronomic prospective is counterproductive as reduction in transpiration is linked with reduced biomass accumulation. Thus, increased emphasis was laid on water-use efficiency.

Physiologically, WUE is defined as the biomass accumulated to total water transpired during the same period or as the ratio of μmoles of CO_2_ assimilated to the moles of water transpired through open stomata. While the CO_2_ partial pressure gradient between atmospheric air (*P*_a_) and intercellular space (*P*_i_) determines photosynthesis, the vapor pressure gradient between leaf and air drives transpiration. On any given day, vapor pressure deficit (VPD) is several orders of magnitude greater than CO_2_ partial pressure gradient resulting in an overwhelmingly higher loss of water through transpiration than carbon assimilated. Therefore, WUE plays an extremely important role in crop water productivity. These facts indicate that further improvement in productivity would heavily relay on the ability to increase the efficiency of resource use. Therefore, water-use efficiency emerges as an important physiological parameter that merits exploitation in order to enhance crop yield under water limited conditions.

## Why breeding for WUE has not been very successful?

Most often, increase in WUE is coupled with the reduction in transpiration, Because of the strong link between transpiration and biomass accumulation, an increase in WUE is often associated with reduced growth rates and yield (Blum, 2009, 2011). This yield reduction while selecting for high WUE distracted breeders from using the trait in their breeding programs. It is well-established that stomata close when plants experience drought either in the soil (Turk et al., 1980) or in air (El-Sharkawy et al., 1984). The regulation of stomatal movement by the plant hormone abscisic acid (ABA) and the control of transpiration is well-studied (Daniel, 1980; Negin and Moshelion, 2016). Plants with reduced stomatal density recorded lower transpiration and hence a higher WUE (Franks et al., 2015), illustrating the role of reduced transpiration in maximizing WUE. When stomata are closed, though would reduce transpiration, a concomitant reduction in CO_2_ entry results in a decrease in photosynthetic carbon assimilation. Thus, an inverse relationship between WUE and biomass accumulation is often noticed (Sheshshayee et al., 2003; Condon et al., 2004). However, a few contradictory evidences have also been reported. Vadez et al. (2011) opined that reduced stomatal conductance and a lower transpiration would save soil moisture that can be effectively utilized during the reproductive stage of crop.

More recently, Hughes et al. (2017) overexpressed *HvEPF1* a transcription factor involved in regulating epidermal pattern to reduce stomatal density, thus contributing to lower transpiration. The maintenance of higher soil moisture levels delayed the stress effects on photosynthesis and observed no reduction in yield while improving WUE. This saving of water for its use at later growth stages would certainly enhance the effective use of water (Blum, 2009), provided direct soil evaporation is minimized. The wheat improvement program of Australia successfully achieved the goal by selecting for higher WUE (Richards, 2000). However, when the soil moisture status improved due to rains during the cropping season, the relative advantage of high WUE cultivars decreased (Richards, 2000). These observations of reduction in yield while selecting for high WUE was the major reason for the unsuccessful attempts in breeding for higher WUE. On the other hand, if carbon assimilatory capacity determines differences in WUE, selection for high WUE does not associate with reduced biomass accumulation (Sheshshayee et al., 2006). These capacity types are characterized by a higher WUE despite relatively higher transpiration.

## WUE is useful only in the background of moderate water use

The model proposed by Passioura (1986), suggests that at a given water use, a genotype that uses water effectively would accumulate more biomass. Though this is intuitively acceptable, due to the tradeoff between water use and WUE, increasing WUE was viewed with a lot of skepticism. To break this impasse, a thorough analysis of the physiological factors that regulate WUE would be necessary.

Two dynamic physiological processes, namely photosynthesis and stomatal conductance govern variability in WUE. Decrease in stomatal conductance restricts transpiration and hence would enhance WUE. Though these “water savers” are relevant under drought conditions, such types, often called “conductance types,” are counterproductive.

On the other hand, if photosynthetic carbon assimilatory capacity determines differences in WUE, selection for higher WUE will not be associated with any reduction in biomass accumulation (Ashok et al., 1999; Sheshshayee et al., 2013). Such types referred to as “capacity types” are most preferred to sustain productivity of crop plants under water limited conditions.

Capacity types are characterized by higher WUE despite a moderately high transpiration. In such types, interdependence between water use and WUE is broken and selection for high WUE does not result in reduced biomass. In other words the relevance of WUE can be fully exploited only when genotypes with high WUE are selected without a substantial reduction in transpiration.

**Figure 2:**
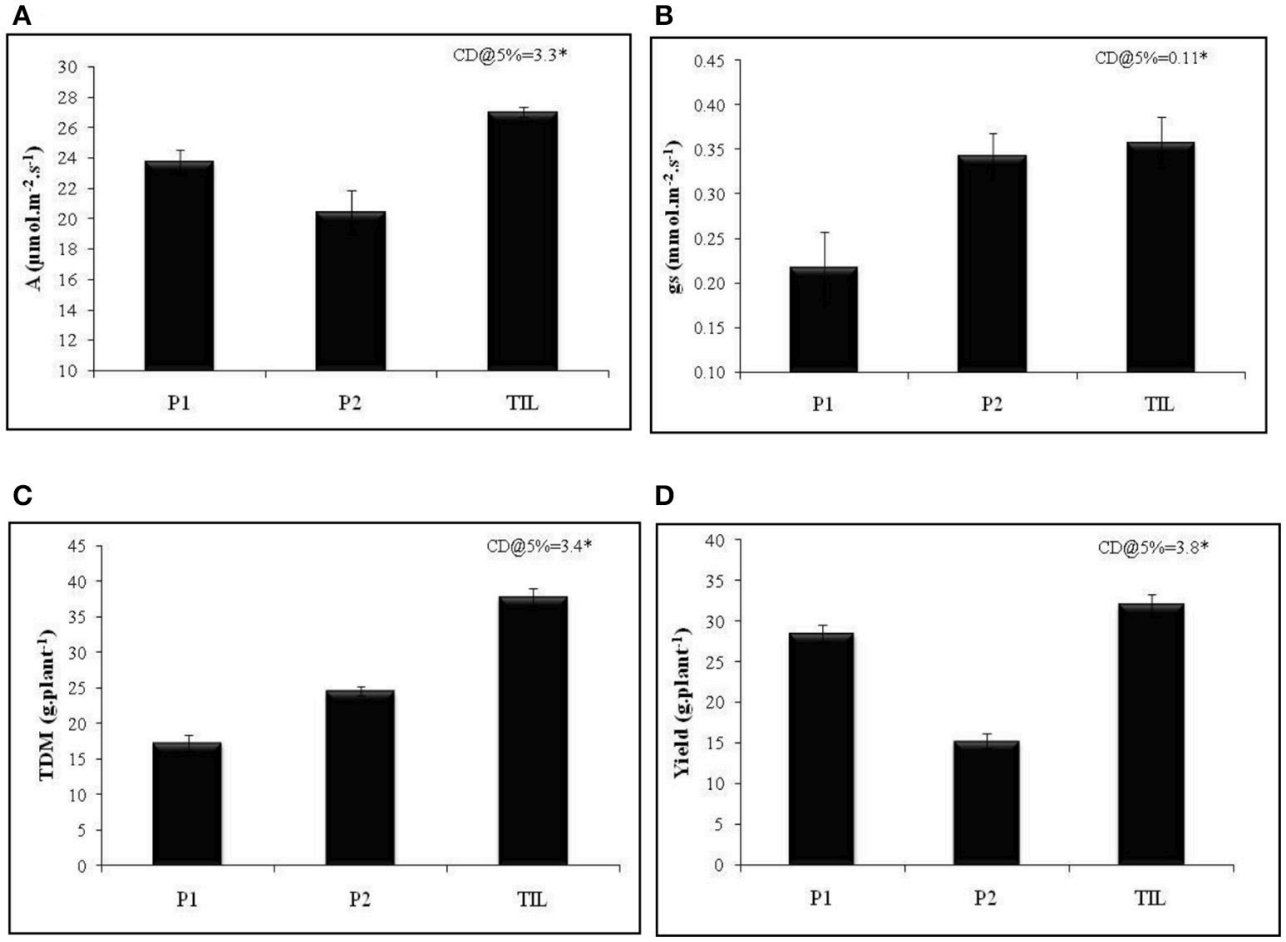
Performance of trait introgressed line KMP175 in aerobic condition **(A)** Photosynthetic rate (*A*), **(B)** Stomatal conductance (*g*_s_), **(C)** Total dry matter (TDM), and **(D)** Yield (g.plant^−1^). P1, Parent 1 (IET 15963); P2, Parent 2 (Thanu); TIL, Trait introgressed line (KMP175). Adopted from Preethi (2013).

To achieve this, traits that promote transpiration and WUE need to be incorporated into the breeding schemes. Deep and extensively developed root system has long been regarded as a potential trait that helps in harnessing water from deeper profiles of soil and hence imparts drought tolerance (Liu et al., 2014). To provide a proof-of-concept that WUE is relevant when simultaneously selected for reasonably high water use, we crossed a rice genotype with superior root trait viz., IET 15963 with a cultivar Thanu that had higher WUE. The segregating lines were advanced to develop a recombinant inbred lines population (RILs) which was extensively phenotyped for roots, WUE and yield traits both under irrigated and aerobic conditions (Raju et al., 2016). Generally, trait introgressed lines had higher total biomass and yield compared to other recombinant lines that had any one of these two traits (data not shown). One of the best trait introgressed line (TIL), KMP175, had superior photosynthetic rate and stomatal conductance compared with the parents (Figures 2A,B). The higher total dry matter accumulation and yield of TIL was significantly higher than the parental lines (Figures 2C,D). This example of trait introgression and several others described above clearly illustrates two important points: (a) WUE is an important trait that holds tremendous promise in improving productivity under drought and (b) the relevance of WUE is noticed only when it is selected along with a reasonably high water use.

## Can genomics accelerate improving drought adaptation?

With the advent of physiological breeding, crop improvement efforts are increasingly becoming dependent on the adoption of molecular and biotechnological approaches (Rosa et al., 2013). A significant reduction in the cost of genome sequencing and molecular characterization of mapping population and germplasm accessions, the discovery of gene and QTL that govern the variations in drought adaptive traits have become affordable. The NGS era has shifted the fragment based analysis of molecular diversity to sequence based approaches. Improvements in high throughput genotyping approaches for sequence based polymorphism (SNP) such as Taq Man (https://www.thermofisher.com/in/en/home/life-science/pcr/real-time-pcr/real-time-pcr-assays.html) and KASP assays (Competitive Allele specific PCR) are among the most commonly adapted approaches. Multiplexing the genome diversity analysis has led to the development of capacities for analyzing over a million variation in a single run (Wang et al., 2009).

Though very high throughput, these marker based molecular characterization has a few lacunae. Development of an array of informative markers may require enormous initial investment for discovery and validation. The markers may display a bias for parental lines leading to low dependability. Thus, the focus is rapidly shifting to whole genome sequencing of an entire set of the population to obtain an unbiased set of SNPs. Approaches such as Restriction Site Associated Sequencing (RAD) and Genotyping-by-Sequencing (GBS) are commonly being adopted (Miller et al., 2007; Elshire et al., 2011). Nevertheless, success in a comprehensive improvement in drought adaptation is still elusive.

## Comprehensive strategies for QTL discovery and introgressing drought adaptive traits

Ever since the first rice genome mapping, sequence information of diverse rice varieties are piling up in the genome databases. Accessibility of high throughput genotyping further resulted in the increased number of linkage maps and genetic stocks in rice (McCouch et al., 2002; Fischer et al., 2012) and other species including semi-perennial species like Mulberry (Mathithumilan et al., 2013, 2016). These technological advances are strongly augmenting the efforts of improving drought adaptation in crop plants through assisting discovery of QTL and genes for drought adaptive traits.

Two approaches, viz., linkage mapping using bi-parental mapping populations and Genome wide association studies (GWAS) using a panel of unrelated germplasm accessions (Yu and Buckler, 2006; Rafalski, 2010; Kikuchi et al., 2017). The linkage mapping approach uses a mapping population developed by crossing contrasting parents, differing in specific drought adaptive traits. The population is extensively phenotyped in target environments and genotyped with molecular markers. Analysis of the co-segregation of marker loci and the trait in question leads to the discovery of QTL by, preferably, composite interval mapping (CIM) strategy. The second approach involves discovery of QTL based on population genetics. This approach involves the phenotypic and molecular characterization of a “panel” of diverse germplasm accessions. This approach relies on the linkage disequilibrium of any pair alleles in the population, and since the germplasm accession represent almost infinite meiotic recombination, the LD based “Association mapping” will have greater resolution. QTL discovery for diverse traits can be simultaneously achieved by this approach and hence this strategy is more robust for discovering QTL for complex physiological traits governing drought adaptation (Raju et al., 2016). Though a large number of published data are available on drought related candidate genes and QTLs (http://www.plantstress.com/), validation of such linked markers and genes are still in the preliminary stage and are debatable (Pennisi, 2008). In spite of such great efforts, development of drought tolerant varieties have not been achieved purely using genomics approaches.

The major factors limiting the precision of QTL localization is the number of progenies used in the study and the inability to accurately phenotype for the specific drought adaptive traits. Further, estimated effects of the identified QTLs are often inconsistent in different genetic backgrounds. An over estimation of the QTL effects in limited progenies leads to wrong predictions, a phenomenon called the “Beavis effect” (Xu, 2003). Thus, the challenge for molecular breeders is to discover heritably stable major QTLs that function independent of genetic backgrounds and to develop an effective breeding method for exploiting of such QTLs (Kearsey and Farquhar, 1998). With the dramatic progress in high throughput phenotyping (Fahlgren et al., 2015) and use of image based trait assessment methods, QTL discovery has now became robust and more accurate. The markers associated with these QTL are expected to strongly complement selection of complex traits and accurate crop improvement (Tanger et al., 2017).

## Translational activities to improve drought adaptation in crop plants

### Physiological breeding

This option of breeding combines the advantages of robust phenotyping for the target traits and the markers associated with these traits. A concurrent selection with both these techniques is expected to ensure success in breeding for enhancing drought adaptation Reynolds and Langridge (2016) elegantly outlined the urgent need and the intricacies of a “Physiological Breeding” approach. This approach relies initially on the identification of appropriate traits that would sustain growth and productivity under resource limiting condition. Therefore, the success of physiological breeding depends on the identification of the most relevant traits and their characterization through appropriate and realistic treatment to mimic field conditions. Significant progress is being achieved through the adoption of molecular tools in introgressing specific traits to a recipient genetic background. Approaches such as Multi-parent Advanced Generation Inter-Cross (MAGIC) have also been devised to combine distinct traits from diverse donor genotypes. After establishing the relevance of combining traits to enhance the performance of rice under water limited conditions (Raju et al., 2014), an extensive effort was made to discover QTL by association mapping to important drought adaptive traits such as root traits and WUE (Raju et al., 2016). These traits are being introgressed using marker assisted breeding involving multiple trait donor parental lines (Prathibha, 2016).

### Transgenics for improvement of water-use efficiency

Improvement of WUE through the use of transgenic technology is a promising alternative. Several studies conducted globally have elucidated the importance and feasibility of various strategies to engineer plants for improved WUE under stress. These options include overexpression of functional genes, regulatory signaling factors, transcription factors, or modifying the root architecture and stomata. Focus has also been on engineering novel proteins that induce advantageous metabolic engineering leading to improved WUE.

#### Controlling or engineering physiological attributes

##### Roots as a target trait to improve drought adaptation

Studies have demonstrated the involvement of ANGUSTIFOLIA3 (*AN3*) as a regulator of drought tolerance through the repression of a MAPKK called *YODA* (Meng and Yao, 2015). Repression of *AN3* was found to result in improved root traits with respect to root length and number of lateral roots. Maintenance of tissue turgor supported by superior water mining through roots has also been shown to enhance photosynthetic carbon assimilation and hence WUE. This has been demonstrated through the deployment of transcription factors (Vemanna et al., 2016).

Aquaporins, the water channels through the cell membrane are gaining relevance as a possible mechanism to enhance water uptake and transport (Ballesta and Carjal, 2014). Aquaporins are integral membrane proteins belonging to the family of Membrane Intrinsic Proteins (MIPs) and facilitate bi-directional transport of water through biological membranes. Based on the spatial distribution, they are divided into five families (Shivaraj et al., 2017). Plant aquaporin's assume significance in the scenario of drought tolerance as their action is related to the regulation of hydraulic conductivities for a better water uptake ability. The dual activity of water and CO_2_ transport, as well as tight cell osmoregulation under water stress, has identified aquaporin's as plausible candidate genes to engineer plants for the improvement drought adaptation. Several genetic engineering studies have demonstrated the utility of aquaporins and suggested them as feasible targets for drought stress mitigation. The over-expression studies demonstrated that the transgenic plants possessed increased root biomass and improved root hydraulic conductance as well as cell hydraulic conductivity. The plants with specific aquaporins were seen to have lower water loss due to effectively managed transpiration and increased water potential and stomatal control (Ballesta and Carjal, 2014).

##### Improvement of intrinsic water use efficiency

Studies have demonstrated that engineering the stomatal density can be a pertinent option to improve photosynthetic CO_2_ fixation and WUE. These anatomical variations have been made possible by identification of regulatory genes like, SDD (stomatal density and distribution). This gene is a subtilisin peptidase that negatively regulates the differentiation of meristemoids and guard mother cells (Franks et al., 2015). Over-expression of this gene resulted in reduced stomatal density leading to reduced transpiration and improved WUE. Several other factors like *EPF, EPFL, YODA*, and *TMM* have been identified and validated which demonstrates the possibility of anatomical modulation of stomata and guard cells to improve WUE.

Improvement of WUE has also been achieved as a result of modulation of leaf surface cuticular wax. It is a well-understood and demonstrated phenomenon that increased epicuticular wax can enhance drought tolerance through water conservation. Studies have shown that over-expression of various transcriptional regulators of biosynthesis of cuticle components resulted in better WUE due to the reduction in water loss (Ruggiero et al., 2017).

Another credible candidate for improved WUE is leaf morphology, especially leaf hairs. A very recent study has identified and validated the use of BLANKET LEAF (a hairy leaf gene) from *Oryza nivara* as a candidate gene for improved photosynthetic WUE (Hamaoka et al., 2017) in rice. This study not only shows the utility of morphological traits for improvement of WUE but also that the crop wild relatives can be exploited as resources for the improvement of various agronomic traits like WUE.

### Improved cellular level tolerance for better WUE

Cellular level tolerance (CLT) is classified as an acquired tolerance trait and hence has great relevance in providing intrinsic tolerance to the plants. Besides several mechanisms, cellular level tolerance generally involves management of free radicals following stress, ionic and osmotic homeostasis, protein folding, and turnover and regulation of cell cycle. CLT therefore deals with sustaining protein synthesis and consequent structural conformation for the maintenance of metabolic activities during stress. Improvement of CLT leading to improved tolerance to stress and WUE has been demonstrated by focusing on regulatory proteins such as transcription factors, protein kinases, helicases, hormones, and other signal molecules; LEA proteins and chaperones. The regulatory proteins control the expression of downstream functional proteins under stress for improved tolerance to stress and WUE (Han et al., 2013). The roles of many transcription factors have been elucidated under stress and they mostly belong to the family of *AP2*/ERF, b*ZIP, NAC, MYB, C2H2* zinc finger, and *WRKY* (Todaka et al., 2015). These are mostly involved in the expression of downstream proteins or in the control of anatomical attributes like stomata leading to improved performance under drought. Further, transcription activators like the DEAD box helicases which are both RNA and DNA-specific have also demonstrated their role in the improvement of WUE by acting at the level of sustaining protein synthesis under stress (Shivakumara et al., 2017).

Plants have evolutionarily developed a variety of signaling networks to adapt to various stresses through morphological, physiological, and metabolic changes (Rejeb et al., 2016). It is a well-known scenario that during stress, proteins on membranes act as receptors, and sensors in transducing the information to their target proteins in the cytoplasm *via* processes such as phosphorylation. A number of signaling factors that operate in response to drought stress have been identified which can be used for deployment into crops through transgenics. Major signaling factors involved in drought mitigation belong to the families of mitogen activated proteins kinases (MAPKs), Ca^2+^ sensing calcium-dependent protein kinases (CDPKs), and signal transduction systems involved in phospholipid metabolism. Several over-expression studies in economically important crops have demonstrated the usefulness of these genes in extenuating drought stress (Rejeb et al., 2016). The plants with ectopic expression of these factors not only showed improved drought tolerance but also improved seed germination and anti-oxidant capacity under drought. As another important aspect, water scarcity is known to increase the levels of ABA content of plant tissues (Davies and Zhang, 1991) which acts as an important messenger in the regulation of plant water status during drought (Tuteja, 2007; Osakabe et al., 2014). ABA-responsive signaling and transcription factors have been identified in the alleviation of drought stress (Tuteja, 2007) making them as alternate options for improved drought tolerance and water-use efficiency.

## Future projections

Global climate change is expected to adversely impact water availability for agriculture in most parts of the world. A comprehensive strategy needs to be evolved and adopted to save water as well as sustaining or even improving productivity under rainfed conditions.

Recent evidences from the literature emphasizes upon the adaptation of a focused trait based crop improvement approach. It is envisaged that selection for trait would lead to a more stable improvement in productivity under diverse environments. This led to the enumeration and classification of drought adaptive traits. Among a number of constitutive and acquired traits those that help to maintain positive tissue turgor and positive carbon gain have great relevance. Water-use efficiency, the ratio of biomass produced to water transpired, is a trait that connects water relations and carbon assimilation traits. These two physiological mechanisms are in turn regulated by stomatal movement which optimizes assimilation and transpiration (Von Caemmerer et al., 2004; Buckley, 2017). While light intensity modulates both carbon assimilation and transpiration, leaf to air VPD drives transpiration. Semi-irrigated aerobic fields are characterized by high VPD which results in substantial volumes of water transpired unproductively (Matthews et al., 2017). Thus, there is a renewed interest in determining the diurnal water loss patterns, especially the “Nocturnal” transpiration. Our recent observations reveal that around 80–100 mL of water is lost from each rice plant during the night periods. This would amount to a significantly large volume of water lost absolutely unproductively. Therefore, WUE computed using water transpired during the entire day may not be realistic. Besides providing a hydrophobic layer on the leaf surface, waxes are known to reflect infrared radiations and hence keep the canopy cooler (Richards et al., 1986; Prathibha, 2013; Boyer, 2015).

Improving photosynthetic efficiency is emerging as yet another powerful approach to improve WUE. Among a number of possible mechanisms, increasing mesophyll conductance (*g*_m_) to CO_2_ transfer is expected to strongly favor improving WUE (Flexas, 2016). A plant with higher g_m_ can afford to maintain a lower g_s_ and thus can reduce transpiration leading to higher WUE (Soolanayakanahally et al., 2009). Similarly, carboxylation efficiency of RuBisCO is an important determinant of creating a diffusion gradient between sub-stomatal cavity and chloroplast (Flexas, 2016). A higher g_m_ coupled with superior carboxylation efficiency can be considered as “capacity types.” These types would maintain higher crop growth rates while selecting for higher WUE.

Such genotypes would eventually have higher yields under water limited conditions. Since carbon assimilatory capacity determines WUE, such plants can be useful even under well-watered conditions.

## Author contributions

SS and RaS conceived the topic and wrote the manuscript; PV generated results for Figure 2 and drafted manuscript with inputs from SS; RoS wrote the transgenics section of the review; SR, SA, PB and PD reviewed the literature and edited the manuscript.

### Conflict of interest statement

The authors declare that the research was conducted in the absence of any commercial or financial relationships that could be construed as a potential conflict of interest. The reviewer, PD, declared a shared affiliation, though no other collaboration, with one of the authors, RoS, to the handling Editor.

## References

[B1] Agrama H. A. S. Zakaria A. G. Said F. B. Tuinstra M. (1999). Identification of quantitative trait loci for nitrogen use efficiency in maize. Mol. Breed. 5, 187–195.

[B2] Alberte R. S. Fiscus E. L. Naylor A. W. (1975). The effects of water stress on the development of the photosynthetic apparatus in greening leaves. Plant Physiol. 55, 317–321. 16659074 10.1104/pp.55.2.317 PMC541607

[B3] Andersen P. Peter B. R. Hazell (1985). The impact of the green revolution and prospects for the future. Food Rev. Int. 11, 1–25.

[B4] Araus J. L. Slafer G. A. Reynolds M. P. Royo C. (2002). Pleant breeding and drought in C_3_ cereals: what should we breed for? Ann. Bot. 89, 925–940. 10.1093/aob/mcf049 12102518 PMC4233799

[B5] Araus J. L. Slafer G. A. Royo C. Serret M. D. (2008). Breeding for yield potential and stress adaptation in cereals. Crit. Rev. Plant Sci. 27, 37–41. 10.1080/07352680802467736

[B6] Araus L. Cairns J. E. (2014). Field high-throughput phenotyping : the new crop breeding. Trends Plant Sci. 19, 52–61. 10.1016/j.tplants.2013.09.008 24139902

[B7] Arora V. K. Singh C. B. Sindhu A. S. Thind S. S. (2011). Irrigation, tillage, mulching effects on soyabean yield and water productivity in relation to soil texture. Agric. Water Manag. 4, 563–568. 10.1016/j.agwat.2010.10.004

[B8] Ashok I. S. Hussein A. Prasad T. G. UdayaKumar M. Nageswara Rao R. C. Wright G. C. (1999). Variation in transpiration efficiency and carbon isotope discrimination in cowpea (*Vigna unguiculata* L. Walp.) genotypes. Aust. J. Plant Physiol. 21, 507–516

[B9] Ballesta M. C. M. Carjal M. (2014). New challenges in plant aquaporin biotechnology. Plant Sci. 217–218, 71–77. 10.1016/j.plantsci.2013.12.006 24467898

[B10] Basu S. Ramegowda V. Kumar A. Pereira A. (2016). Plant adaptation to drought stress. F1000 Res. 5, 1–10. 10.12688/f1000research.7678.1 27441087 PMC4937719

[B11] Blum A. (1996). Constitutive traits affecting plant performance under stress, in Proceedings of a Symposium, CIMMYT (Mexico: CIMMYT).

[B12] Blum A. (2009). Effective use of water (EUW) and not water-use efficiency (WUE) is the target of crop yield improvement under drought stress. Field Crops Res. 112, 119–123. 10.1016/j.fcr.2009.03.009

[B13] Blum A. (2011). Plant Breeding for Water-Limited Environments. New York, NY: Springer Publishing.

[B14] Boyer J. S. (2015). Turgor and the transport of CO_2_ and water across the cuticle (epidermis) of leaves. J. Exp. Bot. 66, 2625–2633. 10.1093/jxb/erv065 25737532 PMC4672191

[B15] Buckley T. N. (2017). Modeling stomatal conductance. Plant Physiol. 117, 572–582. 10.1104/pp.16.01772 PMC5462010 28062836

[B16] Centritto M. Lauteri M. Monteverdi M. C. Serraj R. (2009). Leaf gas exchange, carbon isotope discrimination, and grain yield in contrasting rice genotypes subjected to water deficits during the reproductive stage. J. Exp. Bot. 60, 2325–2339. 10.1093/jxb/erp123 19443613

[B17] Chandra B. R. (2010). Breeding for drought resistance in rice: an integrated view from physiology to genomics. Electron. J. Plant Breed. 1, 1133–1141.

[B18] Condon A. G. Richards R. A. Rebetzke G. J. Farquhar G. D. (2004). Breeding for high water-use efficiency. J. Exp. Bot. 55, 2447–2460. 10.1093/jxb/erh277 15475373

[B19] Daniel C. W. (1980). Biochemistry and physiology of abscisic acid. Ann. Rev. Plant Physiol. 31, 453–489. 10.1146/annurev.pp.31.060180.002321

[B20] Daryanto S. Wang L. Jacinthe P. (2017). Global synthesis of drought effects on cereal, legume, tuber and root crops production : a review. Agric. Water Manag. 179, 18–33. 10.1016/j.agwat.2016.04.022

[B21] Davies W. J. Zhang J. (1991). Root signals and the regulation of growth and development of plants in drying soil. Annu. Rev. Plant Physiol. Plant Mol. Biol. 42, 55–76. 10.1146/annurev.pp.42.060191.000415

[B22] El-Sharkawy M. A. Cock J. H. Held A. A. K (1984). Water efficiency of cassava. II. differing sensitivity of stomata to air humidity in cassava and other warm-climate species. Am. Soc. Agron. 24, 503–507.

[B23] Elshire R. J. Glaubitz J. C. Sun Q. Poland J. A. Kawamoto K. Buckler E. S.. (2011). A robust, simple genotyping-by-sequencing (GBS) approach for high diversity species. PLoS ONE 6:e19379. 10.1371/journal.pone.0019379 21573248 PMC3087801

[B24] Fahlgren N. Gehan M. A. Baxter I. (2015). Lights, camera, action: high-throughput plant phenotyping is ready for a close-up. Curr. Opin. Plant Biol. 24, 93–99. 10.1016/j.pbi.2015.02.006 25733069

[B25] Farooq M. Hussain M. Siddique K. H. M. (2014). Critical reviews in plant sciences drought stress in wheat during flowering and grain- filling periods. Crit. Rev. Plant Sci. 33, 37–41. 10.1080/07352689.2014.875291

[B26] Finlayson C. (2014). The Improbable Primate: How Water Shaped Human Evolution. New York, NY; Oxford: Oxford University Press.

[B27] Fischer K. S. Fukai S. Kumar A. Leung H. Jongdee B. (2012). Field phenotyping strategies and breeding for adaptation of rice to drought. Front. Physiol. 3:282. 10.3389/fphys.2012.0028 22934036 PMC3429056

[B28] Fischer R. A. Byerlee D. Edmeades G. O. (2014). Crop Yields and Global Food Security—Will Yield Increase Continue to Feed the World? Canberra, ACT: Australian Centre for International Agricultural Research Available online at: http://aciar.gov.au/publication/mn.

[B29] Flexas J. (2016). Plant science genetic improvement of leaf photosynthesis and intrinsic water use efficiency in C_3_ plants : why so much little success ? Plant Sci. 251, 155–161. 10.1016/j.plantsci.2016.05.002 27593473

[B30] Franks P. J. Doheny-Adams W. T. Britton-Harper Z. J. Gray J. E. (2015). Increasing water-use efficiency directly through genetic manipulation of stomatal density. New Phytol. 207, 188–195. 10.1111/nph.13347 25754246

[B31] Gaspar M. J. Tania V. Isabel F. Ricardo A. Juan M. (2013). Genetic variation of drought tolerance in *Pinus pinaster* at Three hierarchical levels: a comparison of induced osmotic stress and field testing. PLoS ONE 8:e79094. 10.1371/journal.pone.0079094 24223885 PMC3815124

[B32] Gupta A. K. (2004). Origin of agriculture and domestication of plants and animals linked to early Holocene climate amelioration. Curr. Sci. 87, 54–59. Available online at: http://www.jstor.org/stable/24107979

[B33] Hamaoka N. Yasui H. Yamagata Y. Inoue Y. Furuya N. Araki T.. (2017). A hairy-leaf gene, *BLANKET LEAF*, of wild *Oryza nivara* increases photosynthetic water use efficiency in rice. Rice 10:20. 10.1186/s12284-017-0158-1 28500411 PMC5429320

[B34] Han X. Tang S. An Y. Zheng D. C. Xia X. L. Yin W. L. (2013). Overexpression of the poplar NF-YB7 transcription factor confers drought tolerance and improves water-use efficiency in *Arabidopsis*. J. Exp. Bot. 64, 4589–4601. 10.1093/jxb/ert262 24006421 PMC3808328

[B35] Hughes J. Christopher H. Christian D. Jessica A. D. Lee H. Jennifer S.. (2017). Reducing stomatal density in barley improves drought tolerance without impacting on yield. Plant Physiol. 174, 776–787. 10.1104/pp.16.01844 28461401 PMC5462017

[B36] Impa S. M. Nadaradjan S. Boominathan P. Shashidhar G. Bindumadhava H. Sheshshayee M. S. (2005). Carbon isotope discrimination accurately reflects variability in WUE measured at a whole plant level in rice. Am. Soc. Agron. 45, 2517–2522 10.2135/cropsci2005.0119

[B38] Kamoshita A. Babu R. C. N. Boopathi M. Shu F. (2008). Phenotypic and genotypic analysis of drought-resistance traits for development of rice cultivars adapted to rainfed environments. Field Crops Res. 109, 1–23 10.1016/j.fcr.2008.06.010

[B39] Kearsey M. J. Farquhar A. G. L. (1998). QTL analysis in plants. Where are we now? Heredity 80, 137–142. 10.1046/j.1365-2540.1998.00500.x 9503632

[B40] Khan H. R. Paull J. G. Siddique K. H. M. Stoddard F. L. (2010). Faba bean breeding for drought affected environment: a physiological and Agronomic prospection. Field Crops Res. 115, 279–286. 10.1016/j.fcr.2009.09.003

[B41] Kholová J. Hash T. Kakkera A. Kocova M. Vadez V. (2010). Constitutive water-conserving mechanisms are correlated with the terminal drought tolerance of pearl millet [*Pennisetum glaucum* (L.) R. Br.]. J. Exp. Bot. 61, 369–377. 10.1093/jxb/erp314 19861657 PMC2803210

[B42] Kikuchi S. Bheemanahalli R. Jagadish K. S. V. Kumagai E. Masuya Y. Kuroda E.. (2017). Genome-wide association mapping for phenotypic plasticity in rice. Plant Cell Environ. 40, 1565–1157. 10.1111/pce.12955 28370170

[B43] Kuijken R. C. P. van Eeuwijk F. A. Marcelis L. F. M. Bouwmeester H. J. (2015). Root phenotyping: from component trait in the lab to breeding. J. Exp. Bot. 66, 5389–5401. 10.1093/jxb/erv239 26071534

[B44] Levitt J. (1973). Responses of Plants to Environmental Stresses. 2. New York, NY: Academic press, xiv, 698.

[B45] Liu G. Li X. Jin S. Liu X. Zhu L. Nie Y.. (2014). Overexpression of rice NAC gene SNAC1 improves drought and salt tolerance by enhancing root development and reducing transpiration rate in transgenic cotton. PLoS ONE 9:e86895. 10.1371/journal.pone.0086895 24489802 PMC3904958

[B46] Lopes M. S. Reynolds M. P. (2012). Stay-green in spring wheat can be determined by spectral reflectance measurements (normalized difference vegetation index) independently from phenology. J. Exp. Bot. 63, 3789–3798. 10.1093/jxb/ers071 22412185 PMC3388823

[B47] Manavalan L. P. Nguyen H. T. (2012). Drought tolerance in crops: Physiology to genomics. Plant Stress Physiol. 1:23 10.1079/9781845939953.0001

[B48] Mathithumilan B. Niteen N. K. Jyoti B. Sowmya H. R. Mahadeva A. Madhura J. N.. (2013). Development and characterization of microsatellite markers for Morus spp. and assessment of their transferability to other closely related species. BMC Plant Biol. 13:194. 10.1186/1471-2229-13-194 24289047 PMC3879070

[B49] Mathithumilan B. Sajeevan R. S. Jyoti B. Madhuri T. Karaba N. N. Sheshshayee M. S. (2016). Development and characterization of genic SSR markers from indian mulberry transcriptome and their transferability to related species of Moraceae. PLoS ONE 11:e0162909 10.1371/journal.pone.0162909 27669004 PMC5036888

[B50] Matthew P. R. Maarten V. J. Jean-Marcel R. (2000). Avenues for genetic modification of radiation use efficiency in wheat. J. Exp. Bot. 51, 459–473. 10.1093/jexbot/51.suppl_1.459 10938854

[B37] Matthews J. S. A. Vialet-Chabrand S. R. M. Lawson T (2017). Diurnal variation in gas exchange: the balance between carbon fixation and water loss. Plant Physiol. 174, 614–623. 10.1104/pp.17.00152 28416704 PMC5462061

[B51] McCouch S. R. Teytelman L. Xu Y. Lobos K. B. Clare K. Walton M. (2002). Development and mapping of 2240 new SSR markers for rice (*Oryza sativa* L.). DNA Res. 9, 199–207. 10.1093/dnares/9.6.199 12597276

[B52] Meng L. S. Yao S. Q. (2015). Transcription co-activator *Arabidopsis* ANGUSTIFOLIA3 (AN3) regulates water-use efficiency and drought tolerance by modulating stomatal density and improving root architecture by the transrepression of *YODA* (YDA). Plant Biotechnol. J. 13, 893–902. 10.1111/pbi.12324 25599980

[B53] Miller M. R. Atwood T. S. Eames B. F. Johann K. E. Yi-Lin Y. John H. P.. (2007). RAD marker microarrays enable rapid mapping of zebrafish mutations. Genome Biol. 8:R105. 10.1186/gb-2007-8-6-r105 17553171 PMC2394753

[B54] Mir R. R. Zaman-Allah M. Sreenivasulu N. Trethowan R. Varshney R. K. (2012). Integrated genomics, physiology and breeding approaches for improving drought tolerance in crops. Theor. Appl. Genet. 125, 625–645. 10.1007/s00122-012-1904-9 22696006 PMC3405239

[B55] Monneveux P. Jing R. Misra S. C. (2012). Phenotyping for drought adaptation in wheat using physiological traits. Front. Physiol. 3:429. 10.3389/fphys.2012.00429 23181021 PMC3499878

[B56] Negin B. Moshelion M. (2016). The evolution of the role of ABA in the regulation of water-use. Plant Sci. 251, 82–89. 10.1016/j.plantsci.2016.05.007 27593466

[B57] Osakabe Y. Osakabe K. Shinozaki K. Tran L. S. P. (2014). Response of plants to water stress. Front. Plant Sci. 5:86. 10.3389/fpls.2014.00086 24659993 PMC3952189

[B58] Parry M. A. J. Flexas J. Medrano H. (2005). Prospects for crop production under drought : research priorities and future directions. Ann. Appl. Biol. 147, 211–226. 10.1111/j.1744-7348.2005.00032.x

[B59] Passioura J. B. (1983). Roots and drought resistance. Agric. Water Manag. 7, 1–3. 10.1016/0378-3774(83)90089-6

[B60] Passioura J. B. (1986). Resistance to drought and salinity: avenues for improvement. Aust. J. Plant Physiol. 13, 191–201. 10.1071/PP9860191

[B61] Pennisi E. (2008). The blue revolution, drop by drop, gene by gene. Science 320, 171–173. 10.1126/science.320.5873.171 18403686

[B62] Prathibha M. D. (2013). Genetic Variability and Relevance of Epicuticular Waxes in Rice. Physiological Characterization and Differential Expression of a Few Wax Biosynthetic Genes Leading to Allele Mining. Master's thesis, University of Agricultural Sciences, Bangalore.

[B63] Prathibha M. D. (2016). Introgression of Root and Water Use Efficiency Traits byMarker Assisted Backcross Strategy (MABC) in Rice (Oryza sativa L.) and Validation of Progeny through Physiological Characterization. PhD thesis, University of Agricultural Sciences, Bangalore.

[B64] Preethi N. V. (2013). Introgression of Water Use and Water Use Efficiency in Rice (Oryza sativa L.) – the Relevance of DNA Markers and Stable Isotope Ratios as Surrogates. Master's thesis, University of Agricultural Sciences, Bangalore.

[B65] Prosdocimia M. Tarollia P. CerdÃ A. (2016). Mulching practices for reducing soil water erosion: a review. Earth Sci. Rev. 161, 191–203. 10.1016/j.earscirev.2016.08.006

[B66] Rafalski J. A. (2010). Association genetics in crop improvement. Curr. Opin. Plant Biol. 13, 174–180. 10.1016/j.pbi.2009.12.004 20089441

[B67] Raju B. R. Mohankumar M. V. Sumanthkumar K. Rajanna M. P. Udayakumar M. Prasad T. G. (2016). Discovery of QTL for water mining and water use efficiency traits in Rice under water-limited conditions through association mapping. Mol. Breed. 36:35 10.1007/s11032-016-0457

[B68] Raju B. R. Narayanaswamy S. K. Mohankumar M. V. Udayakumar M. Sheshshayee M. S. (2014). Root traits and cellular level tolerance hold the key in maintaining higher spikelet fertility of rice under water limited conditions. Funct. Plant Biol. 41, 930–939. 10.1071/FP13291 32481046

[B69] Ramakrishna A. Hoang M. T. Suhas P. W. Tranh D. L. (2005). Effect of mulch on soil temperature, moisture, weed infestation and yield of groundnut in Northern Vietnam. Field Crops Res. 95, 115–125. 10.1016/j.fcr.2005.01.030

[B70] Ray D. K. Mueller N. D. West P. C. Foley J. A. (2013). Yield trends are insufficient to double global crop production by 2050. PLoS ONE 8:e66428. 10.1371/journal.pone.0066428 23840465 PMC3686737

[B71] Rejeb K. B. Benzarti M. Debez A. Savoure A. Chedly A. (2016). Water stress in plants, in Water Stress and Crop Plants: A Sustainable Approach ed, Ahmad P. (Chichester: John Wiley & Sons, Ltd), 784 10.1002/9781119054450.ch10

[B72] Reynolds M. P. Pask A. J. D. Mullan D. M. (eds.) (2012). Physiological Breeding I: Interdisciplinary Approaches to Improve Crop Adaptation. Mexico: CIMMYT.

[B73] Reynolds M. Langridge P. (2016). Physiological breeding. Curr. Opin. Plant Biol. 3, 162–171. 10.1016/j.pbi.2016.04.005 27161822

[B74] Reynolds M. Tuberosa R. (2008). Translational research impacting on crop productivity in drought-prone environments. Curr. Opin. Plant Biol. 11, 171–179. 10.1016/j.pbi.2008.02.005 18329330

[B75] Richards R. A. (1996). Defining selection criteria to improve yield under drought. Plant Growth Regul. 20, 157–166. 10.1007/BF00024012

[B76] Richards R. A. (2000). Selectable traits to increase crop photosynthesis and yield of grain crop. J. Exp. Bot. 51, 447–458. 10.1093/jexbot/51.suppl_1.447 10938853

[B77] Richards R. A. Rawson H. M. Johnson D. A. (1986). Glaucousness in wheat: its development and effect on water-use efficiency, gas exchange and photosynthetic tissue temperature. Aust. J. Plant Physiol. 13, 465–473. 10.1071/PP9860465

[B78] Rosa M. Pérez-Clemente V. V. Sara I. Z. María F. L. Valeria M. Aurelio G. (2013). Biotechnological approaches to study plant responses to stress. Biomed. Res. Int. 2013:654120 10.1155/2013/654120 23509757 PMC3591138

[B79] Ruggiero A. Punzo P. Landi S. Costa A. Oosten M. J. V. Grillo S. (2017). Improving plant water use efficiency through molecular genetics. Horticulturae 3:31 10.3390/horticulturae3020031

[B80] Schmid M. Davison T. S. Henz S. R. Pape U. J. Demar M. Vingron M.. (2005). A gene expression map of *Arabidopsis thaliana* development. Nat. Genet. 37, 501–506. 10.1038/ng1543 15806101

[B81] Sheshshayee M. S. Bindumadhava H. Rao N. R. Prasad T. G. Udayakumar M. Wright G. C. (2006). Leaf Chlorophyll concentration relates to transpiration efficiency in Peanut. Ann. Appl. Biol. 148, 7–12. 10.1111/j.1744-7348.2005.00033.x

[B82] Sheshshayee M. S. Bindumadhava H. Shankar A. G. Prasad T. G. Udayakumar M. (2003). Breeding strategies to exploit water use efficiency for crop improvement. J. Plant Biol. 30, 253–268.

[B83] Sheshshayee M. S. Mohan K. M. V. Raju B. R. Prathibha M. D. Rajanna M. P. Mohanraju B. (2013). Enhancing water use efficiency besides effective use of water is a potential strategy in developing rice cultivars suitable for semi-irrigated aerobic cultivation, in International Dialogue on Perception Prospects of Designer Rice Society for Advancement of Rice Research, Directorate of Rice Research, (Hyderabad), 261–272.

[B84] Shivakumara T. N. Sreevathsa R. Dash P. K. Sheshshayee M. S. Papolu P. K. Rao U. (2017). Overexpression of Pea DNA Helicase 45 (*PDH45*) imparts tolerance to multiple abiotic stresses in chilli (*Capsicum annuum* L.). Sci. Rep. 7:2760 10.1038/s41598-017-02589-0 28584274 PMC5459802

[B85] Shivaraj S. M. Deshmukh R. K. Rai R. Bélanger R. Agrawal P. K. Dash P. K. (2017). Genome-wide identification, characterization, and expression profile of aquaporin gene family in flax (*Linumusitatissimum*). Sci. Rep. 7:46137. 10.1038/srep46137 28447607 PMC5406838

[B86] Sinclair T. R. Horie T. (1987). Leaf nitrogen, photosynthesis, and crop radiation use efficiency: a review. Am. Soc. Agron. 29, 90–98.

[B87] Soolanayakanahally R. Y. Guy R. D. Silim S. N. Drewes E. C. Schroeder W. R. (2009). Enhanced assimilation rate and water use efficiency with latitude through increased photosynthetic capacity and internal conductance in balsam poplar (*Populus balsamifera* L.). Plant Cell Environ. 32, 1821–1832. 10.1111/j.1365-3040.2009.02042.x 19712064

[B88] Sung D. Kaplan F. Lee K. Guy C. L. (2003). Acquired tolerance to temperature extremes. Trends Plant Sci. 8, 179–187. 10.1016/S1360-1385(03)00047-5 12711230

[B89] Tanger P. Klassen S. Mojica J. P. Lovell J. T. Moyers B. T. Baraoidan M. (2017). Field-based high throughput phenotyping rapidly identifies genomic regions controlling yield components in rice. Sci. Rep. 7:42839. 10.1038/srep42839 28220807 PMC5318881

[B90] Tardieu F. Draye X. and Javaux M. (2017). Root water uptake and ideotypes of the root system: whole-plant controls matter. Vadose Zone J. 16, 1–10. 10.2136/vzj2017.05.0107

[B91] Todaka D. Shinozaki K. Shinozaki Y. (2015). Recent advances in the dissection of drought stress regulatory networks and strategies for development of drought-tolerant transgenic rice plants. Front. Plant Sci. 6:84. 10.3389/fpls.2015.00084 25741357 PMC4332304

[B92] Tuberosa R. (2012). Phenotyping for drought tolerance of crops in the genomics era. Front. Physiol. 3:347. 10.3389/fphys.2012.00347 23049510 PMC3446691

[B93] Turk K. J. Hall A. E. Asbell C. W. (1980). Drought adaptation of cowpea. I. Influence of drought on seed yield. Agron J. 72, 413–420. 10.2134/agronj1980.00021962007200030004x

[B94] Turner N. C. (1986). Adaptation to H_2_O deficit: a changing prospective. Aust. J. Plant Physiol. 13, 175–190. 10.1071/PP9860175

[B95] Turner N. C. Shahal A. Jens D. B. Chaturvedi S. K. Robert J. F. Christiane L.. (2006). Osmotic adjustment in chickpea (*Cicerarietinum* L.) results in no yield benefit under terminal drought. J. Exp. Bot. 58, 187–194. 10.1093/jxb/erl192 17088363

[B96] Tuteja N. (2007). Abscisic acid and abiotic stress signaling. Plant Signal. Behav. 2, 135–138 10.4161/psb.2.3.4156 19516981 PMC2634038

[B97] Uga Y. Sugimoto K. Ogawa S. Rane J. Ishitani M. Hara N.. (2013). Control of root system architecture by DEEPER ROOTING 1 increases rice yield under drought conditions. Nat. Genet. 45, 1097–1102. 10.1038/ng.2725 23913002

[B98] Unger W. U. Jones O. R. (1980). Effect of soil water content and a growing season straw mulch on green sorghum. Am. Soc. Agron. 45, 129–134.

[B99] Vadez V. Jana K. Grégoire H. Uladzimir Z. Gupta S. K. Tom C. H. (2015). LeasyScan: a novel concept combining 3D imaging and lysimetry for high-throughput phenotyping of traits controlling plant water budget. J. Exp. Bot. 66, 5581–5593. 10.1093/jxb/erv251 26034130 PMC4585418

[B100] Vadez V. Krishnamurthy L. Hash C. T. Upadhyaya H. D. Borrell A. K. (2011). Yield, transpiration efficiency, and water-use variations and their inter-relationships in the sorghum reference collection. Crop Pasture Sci. 62, 645–655. 10.1071/CP11007

[B101] Vemanna R. S. Swetha T. N. Sheela S. H. Babitha C. K. Rohini S. Reddy M. K. (2016). Simultaneous expression of regulatory genes associated with specific drought-adaptive traits improves drought adaptation in peanut. Plant Biotechnol. J. 14, 1008–1020. 10.1111/pbi.12461 26383697 PMC11388866

[B102] Venuprasad R. Lafitte H. R. Atlin G. N. (2007). Response to direct selection for grain yield under drought stress in rice. Crop Sci. 47, 285–293. 10.2135/cropsci2006.03.0181

[B103] Vinocur B. Altman A. (2005). Recent advances in engineering plant tolerance to abiotic stress: achievements and limitations. Curr. Opin. Biotechnol. 16, 123–132. 10.1016/j.copbio.2005.02.001 15831376

[B104] Von Caemmerer S. Lawson T. Oxborough K. Baker N. R. Andrews T. J. Raines C. A. (2004). Stomatal conductance does not correlatewith photosynthetic capacity in transgenic tobacco with reduced amounts of Rubisco. J. Exp. Bot. 55, 1157–1166. 10.1093/jxb/erh128 15107451

[B105] Wang G. P. Hui Z. Li F. Zhao M. R. Zhang J. Wang W. (2010). Improvement of heat and drought photosynthetic tolerance in wheat by over accumulation of glycinebetaine. *Plant Biotechnol*. Rep. 4, 213–222. 10.1007/s11816-010-0139-y

[B106] Wang H. H. Farren J. I. Peter A. Zachary Z. S. George X. Craig R. F.. (2009). Programming cells by multiplex genome engineering and accelerated evolution. Nat. Lett. 460, 894–899. 10.1038/nature08187 19633652 PMC4590770

[B107] Xin Z. Aiken R. M. Burke J. J. (2009). Genetic diversity of transpiration efficiency in sorghum. Field Crops Res. 111, 74–80. 10.1016/j.fcr.2008.10.010

[B108] Xu Y. (2003). Theoretical basis of the beavis effect. Genetics 165, 2259–2268. 14704201 10.1093/genetics/165.4.2259 PMC1462909

[B109] Yang W. Duan L. Chen G. Xiong L. Liu Q. (2013). Plant phenomics and high-throughput phenotyping: accelerating rice functional genomics using multidisciplinary technologies. Curr. Opin. Plant Biol. 16, 180–187. 10.1016/j.pbi.2013.03.005 23578473

[B110] Yu J. Buckler E. (2006). Genetic association mapping and genome organization of maize. Curr. Opin. Biotechnol. 17, 155–160. 10.1016/j.copbio.2006.02.003 16504497

[B111] Yusuf K. Christina N. K. (2008). Monitoring the impacts of urbanization and industrialization on the agricultural land and environment of the Torbali, Izmir region, Turkey. Environ. Monit. Assess. 136, 289–297. 10.1007/s10661-007-9684-4 17370130

