# Corrigendum: Introgression of Physiological Traits for a Comprehensive Improvement of Drought Adaptation in Crop Plants

**DOI:** 10.3389/fchem.2018.00382

**Published:** 2018-08-24

**Authors:** Sheshshayee M. Sreeman, Preethi Vijayaraghavareddy, Rohini Sreevathsa, Sowmya Rajendrareddy, Smitharani Arakesh, Pooja Bharti, Prathibha Dharmappa, Raju Soolanayakanahally

**Affiliations:** ^1^Department of Crop Physiology, University of Agricultural Sciences, Bengaluru, India; ^2^ICAR-National Research Centre for Plant Biotechnology, New Delhi, India; ^3^Saskatoon Research and Development Centre, Agriculture and Agri-Food Canada, Saskatoon, SK, Canada

**Keywords:** drought adaptive traits, water productivity, water-use efficiency, physiological breeding, carbon isotope discrimination, cellular level tolerance, transgenics

## Incorrect reference

In the original article, the reference for Matthews et al. (2017) was incorrectly written as Jack et al. (2017). A correction has been made to *FUTURE PROJECTIONS, Paragraph 2*:

Recent evidences from the literature emphasizes upon the adaptation of a focused trait based crop improvement approach. It is envisaged that selection for trait would lead to a more stable improvement in productivity under diverse environments. This led to the enumeration and classification of drought adaptive traits. Among a number of constitutive and acquired traits those that help to maintain positive tissue turgor and positive carbon gain have great relevance. Water-use efficiency, the ratio of biomass produced to water transpired, is a trait that connects water relations and carbon assimilation traits. These two physiological mechanisms are in turn regulated by stomatal movement which optimizes assimilation and transpiration (Von Caemmerer et al., 2004; Buckley, 2017). While light intensity modulates both carbon assimilation and transpiration, leaf to air VPD drives transpiration. Semi-irrigated aerobic fields are characterized by high VPD which results in substantial volumes of water transpired unproductively (Matthews et al., 2017). Thus, there is a renewed interest in determining the diurnal water loss patterns, especially the “Nocturnal” transpiration. Our recent observations reveal that around 80–100 mL of water is lost from each rice plant during the night periods. This would amount to a significantly large volume of water lost absolutely unproductively. Therefore, WUE computed using water transpired during the entire day may not be realistic. Besides providing a hydrophobic layer on the leaf surface, waxes are known to reflect infrared radiations and hence keep the canopy cooler (Richards et al., 1986; Prathibha, 2013; Boyer, 2015).

In addition, the first author's name was repeated twice in the reference list for (El-Sharkawy et al., 1984). The corrected reference appears below.

The authors apologize for this error and state that this does not change the scientific conclusions of the article in any way.

The original article has been updated.

## Conflict of interest statement

The authors declare that the research was conducted in the absence of any commercial or financial relationships that could be construed as a potential conflict of interest.
